# Therapeutic opportunities in EBV-positive gastric cancer subtypes

**DOI:** 10.1177/17588359251396619

**Published:** 2025-12-03

**Authors:** Hasan Al-Sattar, Richard Owen, Amir Mashia Jaafari, Abhishek Saha, Koushikk Ayyappan, Aruni Ghose, Stergios Boussios, Sola Adeleke

**Affiliations:** School of Medicine and Biomedical Sciences, University of Oxford, Oxford, UK; Ludwig Institute for Cancer Research, University of Oxford, Oxford, UK; Ludwig Institute for Cancer Research, University of Oxford, Oxford, UK; Department of Surgery, Churchill Hospital, Oxford University Hospitals NHS Trust, Oxford, UK; School of Medicine and Biomedical Sciences, University of Oxford, Oxford, UK; School of Medicine and Biomedical Sciences, University of Oxford, Oxford, UK; School of Medicine and Biomedical Sciences, University of Oxford, Oxford, UK; Department of Medical Oncology, St Bartholomew’s Hospital, Barts Health NHS Trust, London, UK; Barts Cancer Institute, Cancer Research UK City of London Centre, Queen Mary University of London, London, UK; Department of Medical Oncology, Ioannina University Hospital, Ioannina, Greece; School of Cancer and Pharmaceutical Sciences, King’s College London, London, UK; Department of Medical Oncology, Medway NHS Foundation Trust, Gillingham, UK; School of Biomedical Engineering and Imaging Sciences, King’s College London, London WC2R 2LS, UK; Guy’s Cancer Centre, Guy’s and St. Thomas’ NHS Foundation Trust, London, UK

**Keywords:** EBV, EBV-positive gastric cancer, Epstein–Barr virus, gastric cancer, immune biomarkers, immunotherapy

## Abstract

Immunotherapy has shown inconsistent results in Epstein–Barr virus-associated gastric cancer (EBVaGC) despite being associated with an active tumour microenvironment. This calls for the identification of subtypes within the EBVaGC subtype, and subsequent treatments tailored for their properties. This review identified six different EBVaGC subtypes alongside potential therapeutic opportunities. EBVaGCs, which express immune checkpoints, high microsatellite instability or high tumour mutational burden, are shown to respond better to immune checkpoint inhibitors, each due to their own specific characteristics. Co-infection of EBV and *Helicobacter pylori* in gastric cancer (GC) can exacerbate their impact on inflammatory stress and has the potential to be treated by antiviral agents and antimicrobials. EBVaGCs are also more likely to express wild-type p53 than other GCs, which suggests potential for lytic-induction therapy, where the EBV genome is kicked out of latency and subsequently killed using antiviral nucleoside analogue prodrugs. Lastly, EBVaGC is more likely to express the PI3K and ARID1A mutations, which can potentially be treated using PI3K/mTOR dual inhibitors and Akt/PARP inhibitors. These six subtypes could aid the selection of more successful treatments for EBVaGC, thereby improving the current overall survival and prognosis of patients.

## Introduction

Gastric cancer (GC) is the third most fatal type of cancer globally, with ~780,000 annual deaths.^1^ The Cancer Genome Atlas (TCGA) divides GC into four molecular subtypes: Epstein–Barr virus-associated gastric cancer (EBVaGC), microsatellite instability, genomic stability and chromosomal instability.^2^ EBVaGC makes up 1.3%–30.9% of all GC globally, depending on geographic distribution.^3–5^ EBV infection is confirmed by EBV-encoded small RNA in situ hybridisation testing of GC tissue.^6,7^ One hallmark of EBVaGC has been described as the DNA hypermethylation of tumour suppressor genes and other genes that inhibit metastasis.^8^ Consequently, EBVaGC typically has a more favourable prognosis compared to EBV-negative GC, primarily due to a lower incidence of metastasis to regional lymph nodes.^9^ Additionally, the majority of early-stage EBVaGC cases can be treated with surgical excision without subsequent recurrence.^10^ Most importantly, several studies have shown that patients with EBVaGC reported better outcomes with anti-programmed cell death-ligand 1 (PD-L1) antibody treatments.^11–14^ This may be due to the active immune microenvironment induced by EBV infection,^15^ as EBV-positive status induces a tumour microenvironment (TME) with increased immune cell infiltration and elevated expression of immune response genes.^16–19^ However, the effect of immunotherapy treatment in EBVaGC is inconsistent, and no predictive biomarkers for efficacy have been reported.^17,20^ It is therefore necessary to explore EBV infection-induced changes and their TME to identify potential subtypes within the EBVaGC subtype and, subsequently, decide whether these subtypes should be treated differently.

## Subtypes of EBVaGC and subsequent therapeutic opportunities

### Immunotherapy-responsive EBVaGC subtypes

EBVaGC has been recognised for its immunologically active TME, making it a compelling candidate for immunotherapy. International guidelines such as those from the NCCN and ESMO now recommend immune checkpoint inhibitors (ICIs), particularly anti-PD-1 and anti-PD-L1 antibodies, in advanced GC where predictive biomarkers are present.^21,22^ Notably, subgroups of EBVaGC may demonstrate high PD-L1 expression, high microsatellite instability (MSI-H) or elevated tumour mutational burden (TMB)—all of which are independently associated with improved response to immunotherapy.^23^ However, emerging evidence indicates considerable overlap between these biomarkers.^24,25^ For example, EBVaGC with high PD-L1 expression may also exhibit high TMB or MSI-H status, and some of the underlying genetic drivers may contribute to multiple immunogenic phenotypes simultaneously. This interplay complicates patient stratification and highlights the need for a nuanced approach to treatment selection. While this section acknowledges such overlap, each feature will be discussed individually in the following subsections to clearly delineate its respective therapeutic implications.

#### Subtype I—EBVaGC expressing immune checkpoints

Immune checkpoints refer to molecules that act as immune system regulators, preventing self-tolerance. PD-L1 is an immune checkpoint protein expressed by some tumours that can bind to PD-1 on T cells and other immune cells, leading to exhaustion and suppression of anti-tumour immunity.^26^ A recent meta-analysis of 43 publications comprising 11,327 patients showed an increased association between PD-L1 expression and EBVaGC, supporting the rationale for targeting the PD-1/PD-L1 axis in this subgroup.^27^

Pembrolizumab, an anti-PD-1 antibody, became the first FDA-approved ICI for non-primary tumour-specific use in the treatment of metastatic or unresectable solid tumours.^28^ Its approval for recurrent and metastatic GC followed the promising results of the KEYNOTE-012 trial, where it demonstrated a significant overall response rate after two or more lines of therapy.^29^ Subsequent phase II trial KEYNOTE-059 confirmed pembrolizumab’s effectiveness as monotherapy in third-line treatment for GC with a combined positive score of ⩾1.^30^ Numerous phase III trials have since investigated ICI response in GC (Table 1). Given the documented PD-L1 overexpression in EBVaGC,^31^ ongoing clinical trials are investigating anti-PD-1 drugs like nivolumab (NCT02951091) and avelumab (NCT01772004) in the treatment of EBVaGC.^12,27,32^

**Table 1.** Outcomes of relevant phase III clinical trials for ICI in GC.

Trial name	Phase	Target	Treatment	Control arm	Key result
KEYNOTE-061	III	PD-1	Pembrolizumab	Paclitaxel	Median OS 9.1 vs 8.3 months, median PFS 1.5 vs 4.1 months
KEYNOTE-062	III	PD-1	Pembrolizumab + chemotherapy	5FU or capecitabine and cisplatin, with a placebo	Median OS 12.5 vs 11.1 months, median PFS 6.9 vs 6.4 months
KEYNOTE-063	III	PD-1	Pembrolizumab	Paclitaxel	Median OS 8 vs 8 months, median PFS 2 vs 4 months
KEYNOTE-811	III	PD-1 + HER2	Chemotherapy + trastuzumab and pembrolizumab	5FU and cisplatin, or capecitabine and oxaliplatin, with trastuzumab	OS awaited; ORR, 74.4% vs 51.9%
ATTRACTION-2	III	PD-1	Nivolumab	Placebo	Median OS 5.3 vs 4.1 months, median PFS 1.6 vs 1.5 months
ATTRACTION-4	III	PD-1	Capecitabine or S-1 and oxaliplatin with nivolumab	Capecitabine or S-1 and oxaliplatin	Median OS 17.5 vs 17.2 months, median PFS 10.5 vs 8.3 months
CheckMate 649	III	CTLA-4 + PD-1	Ipilimumab + Nivolumab	CapeOX/FOLFOX	Did not meet the prespecified boundary for significance
Javelin Gastric 100	III	PD-L1	Avelumab	Continuation of FOLFOX or CAPOX	Median OS 10.4 vs 10.9 months, and median PFS 3.2 vs 4.4 months
Javelin Gastric 300	III	PD-L1	Avelumab	Irinotecan or paclitaxel	Median OS 4.6 vs 5.0 months, median PFS 1.4 vs 2.7 months

GC, gastric cancer; ICIs, immune checkpoint inhibitors; OS, overall survival; PD-1, programmed cell death 1.

CAPOX, Capecitabine and oxaliplatin; FOLFOX, Folinic acid, fluorouracil and oxalipatin; PFS, Progression free survival; ORR, Overall response rate.

Derks et al.^33^ revealed that around 15% of EBVaGC cells have gene amplification of chromosome segment 9p24.1, which encodes the ligands for PD-1, explaining the higher expression of PD-1 in EBVaGC. Furthermore, a study involving 61 Korean patients with advanced GC treated with pembrolizumab revealed a significantly higher objective response rate in PD-L1-positive patients (50%) than in PD-L1 negative patients (0%).^34^ This emphasises the potential benefits of using PD-L1 expression as a biomarker for ICI treatment in EBVaGC.

**Figure 1.**
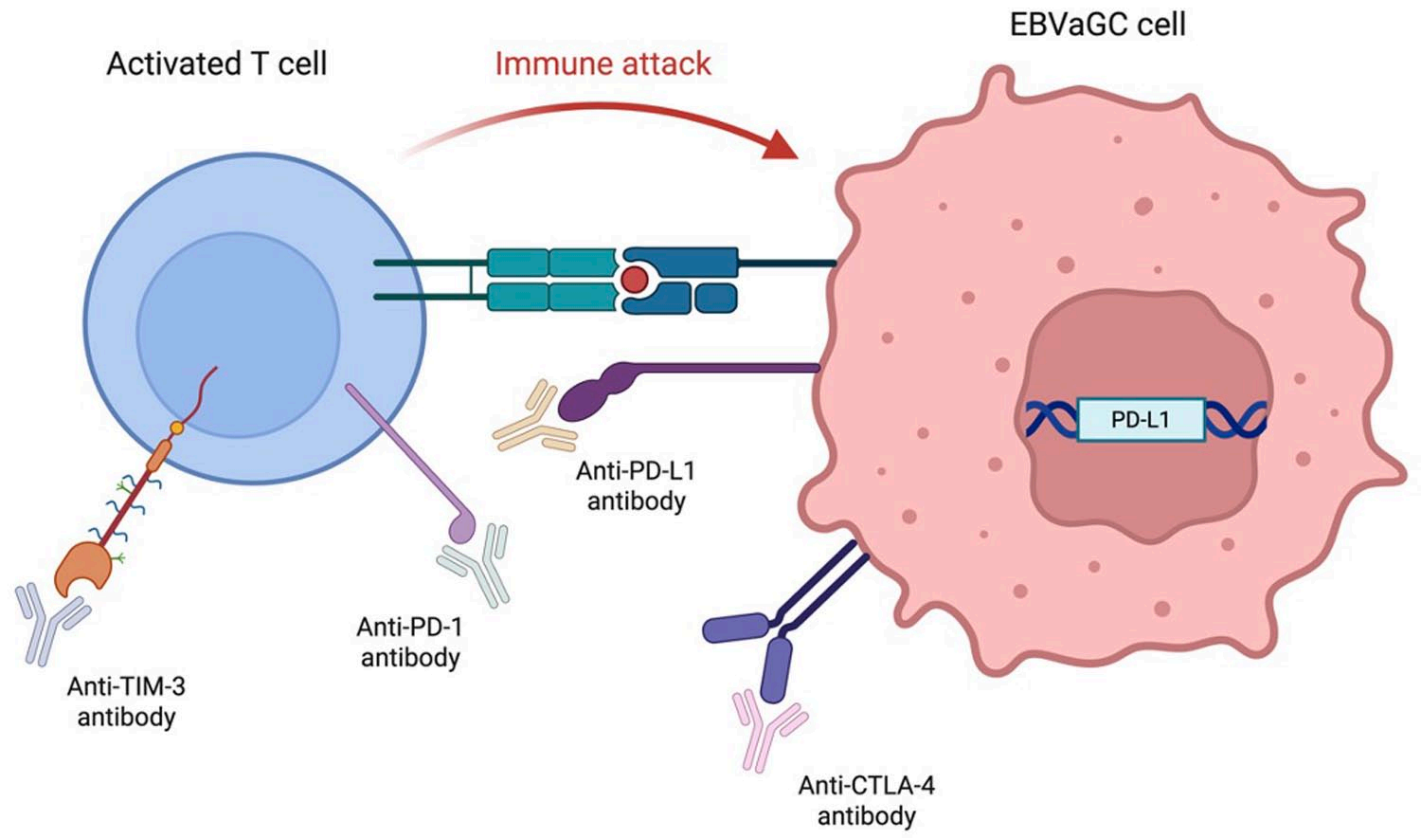
The different opportunities for immune checkpoint blockade for EBVaGC. Source: Figure created with BioRender.com This diagram shows the immune checkpoint molecules (PD-1/PD-L1, CTLA-4, TIM-3) expressed in EBVaGC and the corresponding immune checkpoint inhibitors for targeted immune checkpoint blockade. EBVaGC, EBV-associated gastric cancer; PD-L1, programmed cell death-ligand 1.

A 2022 study investigated differences in the TME between the ICI responders and non-responders in EBVaGC and showed that the density of CTLA-4+ cells and TIM-3+ cells was substantially higher in the non-response group.^35^ CTLA-4, like PD-1, is another immune checkpoint protein found on regulatory T cells, which downregulates the immune response by binding to CD80 or CD86 on antigen-presenting cells.^36^ The study saw that patients with EBVaGC with high CTLA-4 levels were less responsive to anti-PD-1/L1 monotherapy, but demonstrated improved outcomes with combination PD-1/L1 plus CTLA-4 blockade. TIM-3 is another immune checkpoint known for inhibiting the immune response.^37^ A recent study demonstrated that the presence of TIM-3+ cell infiltration is linked to an immunoevasive subtype of GC characterised by CD8+ T-cell dysfunction, highlighting TIM-3 as a promising immunotherapy target for GC.^38^ Additionally, another study showed that combining anti-PD-1 and anti-TIM-3 monoclonal antibodies had an additive effect T-cell cytotoxicity, suggesting that dual ICI targeting PD-1 and TIM-3 could enhance response rates in GC.^39^ Given these findings, triple blockade therapy targeting PD-1, CTLA-4 and TIM-3 could be a rational strategy for benefiting patients with EBVaGC characterised by high densities of CTLA-4+ and TIM-3+ cells (Figure 1). Although this study’s small sample size and homogeneous populations could have limited its results, it was the largest study of its kind to date.

There could also be potential for identifying genes as biomarkers for improved ICI response. One such example is CHAF1A, a well-defined oncogene, identified earlier this year as a novel biomarker for ICI response in EBVaGC.^40^ CHAF1A expression strongly correlates with MSI, TMB and PD-L1 expression, and another study showed that in vivo CHAF1A knockdowns in immunocompetent C57BL/6 mice impaired the effect of an anti-PD-1 antibody.^41^ This indicates that CHAF1A has the potential to be an independent biomarker for improving patient selection for ICI therapy in EBVaGC.

#### Subtype II – MSI-H

MSI-H GC is one of four distinct subtypes of GC set out by the TCGA, alongside EBVaGC. Microsatellites are short tandem repeats distributed throughout the whole genome and are prone to a high mutation rate. Therefore, MSI is defined as a hyper-mutable phenotype at genomic microsatellites in the presence of mismatch repair deficiency (dMMR).^42^ The MMR system is a highly conserved cellular process that identifies and repairs mismatched bases, often arising from errors during DNA replication, genetic recombination or chemical/physical insults.^43^ Deficiency or loss of expression in one or more of its key components leads to impaired repair of DNA mismatches.^44^ This can develop a ‘mutator phenotype’ characterised by numerous frameshift mutations in coding and non-coding microsatellites, as well as at other genetic loci beyond microsatellites. This then leads to the MSI-H phenotype, which is closely associated with the development of hereditary and sporadic tumours.^45^

MSI-H and EBV-positive GCs are thought to be mutually exclusive, with 0% of patients in the SNUH GC cohort and TCGA-STAD study having GC with both MSI-H and EBV.^46,47^ To understand this exclusivity, Kim et al.^48^ generated MSI-H status in the EBV-positive GC cell-line NCC24 by MLH1 knockout via CRISPR-Cas9. These cells displayed decreased stemness both in vitro and in vivo, suggesting that double-positive GC is not observed in clinical settings due to the novel introduction of a DNA MMR gene deficiency negatively affecting tumour stemness when EBV positivity is already present. However, as this study used limited flow cytometry and immunohistochemical markers, the conclusions that can be drawn on the reduction of cancer stem cells caused by the downregulation of MLH1 expression are limited. Future studies could perhaps use cancer stem cell-associated mRNA signatures as an alternative option. Furthermore, results observed in cell lines may not translate in the clinic; a study by Leung et al.^49^ discovered two patients with GCs that were both EBV-positive and MSI-H in a series of 79 cases (18 EBV-positive, 13 MSI-H). Unfortunately, clinical studies comparing these double-positive GC cases to just MSI-H GC or EBVaGC remain limited. A previous study showed that treatment with ICIs in MSI-H GC is linked to a more favourable prognosis.^50^ It remains to be determined whether an increase in mutational load and neoantigens due to MMR deficiency would be advantageous in boosting the responsiveness to immunotherapeutic agents seen in EBVaGC.

#### Subtype III – TMB

TMB is a genomic biomarker calculated by the total number of somatic alterations detected using exome sequencing of the coding region.^51^ Highly mutated tumours can express neoantigens, which cause T-cell activation, suggesting that TMB may be a useful predictive biomarker for ICI response.^52^ Studies have demonstrated that tumours with high TMB showed positive treatment outcomes with PD-1/PDL-1 or CTLA-4 blockade.^53–55^ A pan-tumour analysis of 12 trials using pembrolizumab treatment highlighted that patients with high TMB displayed significant improvement in pembrolizumab efficacy.^56,57^

He et al.^58^ used a next-generation sequencing assay targeting 295 different cancer-related genes in 73 EBVaGC and 75 EBV-negative GC specimens and compared results with overall survival (OS). The study integrated EBV infection with TMB and large genomic instability (LGI) status and identified four distinct molecular subtypes (EBV+/TMB-high, EBV+/TMB-low, EBV-/LGI- and EBV-/LGI+). This approach yielded a significantly different OS for each subtype, with 96.2, 75.3, 44.4 and 20.2 months, respectively. They then used functional annotation and pathway enrichment analysis to explain the mechanism behind the higher OS of the EBV+/TMB-high group. The mutated genes specific to this subtype included genes involved in the JAK/STAT pathway, which have previously been linked with tumour suppression, improved response to immunotherapy and improved prognosis in cancer patients.^59^ The study also demonstrated that the EBV+/TMB-low tumours were characterised by mutations in DNA repair genes and the MMR pathway, which may enhance the tumour’s sensitivity to chemotherapy, thereby improving patient prognosis. Overall, the study highlights the potential use of TMB as a biomarker for EBVaGC, where their respective genetic characteristics may guide different treatment strategies for patients with either high TMB or low TMB.

Analysis from the KEYNOTE-062 trial also indicated an association between TMB and clinical outcomes in gastric/gastroesophageal junction adenocarcinoma patients receiving pembrolizumab-monotherapy and pembrolizumab-chemotherapy as first-line treatment.^60^ However, this association was attenuated after excluding patients with MSI-high tumours, suggesting that TMB may not effectively predict better clinical outcomes. It must be noted, though, that this analysis evaluated TMB in patients with gastric and gastroesophageal junction adenocarcinoma, not EBVaGC, where distinct factors may influence the effect of TMB on clinical outcomes.

Another study used extensive genomic analyses to compare responders and non-responders to ICI in search of potential pretreatment biomarkers for EBVaGC.^35^ They identified that the SMARCA4 mutation might serve as a positive predictor of ICI efficacy in EBVaGC. Previous evidence suggests that improved ICI response in SMARCA4-deficient cancers may be attributed to increased TMB and activation of the TME.^61–63^ This reinforces the notion that EBVaGC patients showing high TMB could be treated as a subtype of EBVaGC in which immunotherapy should cause a better response.

### Subtype IV – *Helicobacter pylori* co-infection

In addition to EBV infection, *Helicobacter pylori* (HP) infection is a significant risk factor in the development of GC and the most common cause of gastric carcinogenesis.^64^ Previous research indicates that EBV and HP coinfection may synergistically induce severe inflammatory responses and increase the risk of GC.^65^ However, it remains unclear whether the malignancy arises from accumulated tissue damage caused by EBV and HP coinfection or the close interaction between EBV and HP genes.^66^

The interaction between the two infectious agents can have oncogenic effects or exacerbate their impact. Several studies have demonstrated that the presence of HP can stimulate the reactivation of EBV from its latent state in gastric epithelial cells,^67^ whilst Saiki et al.^68^ proposed that the inflammatory stress induced by HP may attract more EBV-carrying lymphocytes, increasing the likelihood of epithelial cell infection by these lymphocytes. Cárdenas-Mondragón et al.^65^ discovered that EBV acts as a cofactor in triggering gastric inflammation in conjunction with HP in gastric diseases. Rihane et al.^69^ aimed to assess the prevalence of co-infections in GC tissues. While most cancers typically increase with age, their study revealed a significant correlation between early-onset GC and EBV + HP co-infection, suggesting that co-infection promotes tissue malignancy. Together, these results have prompted suggestions that combining antimicrobials and antiviral agents with traditional cancer therapies may offer a better clinical response in EBVaGC patients with HP infection.

**Figure 2.**
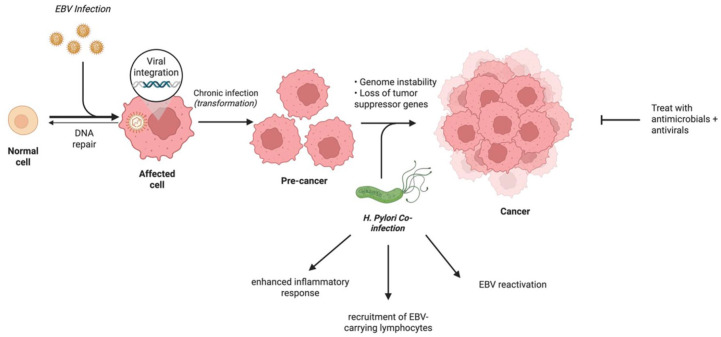
The mechanism of EBVaGC and *Helicobacter pylori* co-infection, and subsequent treatment opportunity. Source: Figure created with BioRender.com This diagram illustrates the interplay between EBV and *H. pylori* co-infection in gastric epithelial cells, highlighting the mechanisms of inflammation and oncogenesis and potential therapeutic strategies involving combined antiviral and antimicrobial agents. EBV, Epstein-Barr virus; EBVaGC, EBV-associated gastric cancer.

While several studies have observed an association between coinfection and GC pathogenesis, few have specifically investigated the impact of EBV and HP coinfection on clinical outcomes and prognosis in GC. Noh et al.^70^ analysed 956 patients who underwent surgery for GC and subdivided them according to morphology, EBV infection and HP infection. Using a median follow-up period of 72 months, the OS was not significantly different between the EBV + HP coinfection group and others (97.6% vs 86.8%, log-rank *p* = 0.144), suggesting that coinfection is not an independent prognostic factor for GC. However, this study did not assess whether the coinfection subgroup should be treated differently; rather, it investigated OS after surgical resection in each subgroup. Further studies should examine whether treatments tailored for EBV + HP GC (such as antiviral and antimicrobials) would have a better prognostic effect than EBVaGC or HP-positive GC alone (Figure 2). There have also been reported cases of these co-infections also harbouring MSI-H, which is a further reminder that these subtypes are not mutually exclusive and that some overlap may exist. In these cases, all these factors must be considered; for example, in this instance, immunotherapy may be another therapeutic option.

### Subtype V – wild-type p53

Approximately 70% of all GC cases are characterised by TP53 mutations,^71^ whereas TP53 mutations in EBVaGC are instead rare, with a mutation rate of only 15.1%.^58,72,73^ The distinct characteristic that EBVaGC mostly expresses, wild-type p53, can be exploited in several ways. Most notably, lytic-induction therapy or ‘kick-and-kill’ involves reactivating EBV in tumour cells from its latent form of infection into its lytic replication cycle. Reactivation initiates with the expression of the EBV-encoded immediate-early proteins BZLF1 and BRLF1.^74^ Previous studies have shown that induction of the BZLF1 gene expression to induce EBV reactivation in EBVaGC cells requires wild-type p53 as a co-activator.^75^ These immediate-early proteins subsequently induce the expression of multiple viral early genes, including BGLF4 and BXLF1, which encode a protein kinase and thymidine kinase, respectively. This activates the EBV lytic-cycle cascade, leading to viral genome replication and the expression of numerous late genes necessary for the production of infectious viral particles and viraemia in the patient.^76^

**Figure 3.**
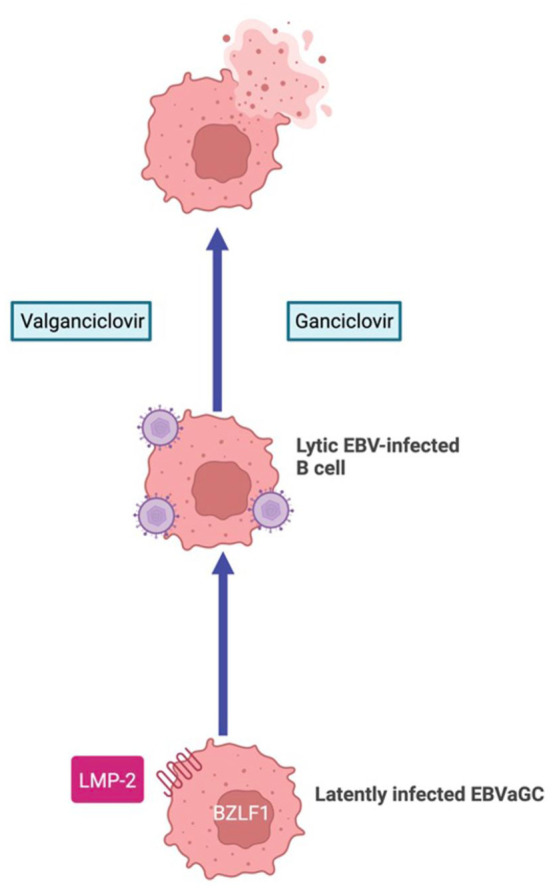
Figure illustrating how selective reactivation and eradication (‘kick and kill’) can be used to specifically target and treat EBVaGC cells. Source: Figure created with BioRender.com This diagram illustrates the ‘kick-and-kill’ or lytic-induction therapeutic strategy in EBVaGC, showing how reactivation of latent EBV within tumour cells allows selective eradication through antiviral nucleoside analogues such as ganciclovir and valganciclovir. EBV, Epstein-Barr virus; EBVaGC, EBV-associated gastric cancer.

Selective eradication of EBV-positive cancer cells and prevention of viraemia can be accomplished by administering an antiviral nucleoside analogue prodrug such as ganciclovir or valganciclovir (Figure 3). Ganciclovir must be converted to its active form through phosphorylation by the viral protein kinase, a kinase expressed only in cells replicating EBV or another herpesvirus.^77^ Phosphorylated ganciclovir is then integrated into DNA as a chain terminator, inhibiting both viral and cellular DNA replication and causing the death of the reactivated EBV-positive tumour cells while preserving most EBV-negative normal tissue. Additionally, cells adjacent to the virally reactivated cells are often eliminated via a phenomenon known as the ‘bystander effect’.^78^ Therefore, it is not essential for lytic reactivation efficiency to reach 100% to eliminate most or all cells in an EBV-positive tumour. Hence, lytic-induction therapy may represent a highly specific approach for targeting EBVaGC.

However, a major concern of lytic-induction therapy is the promotion of viral dissemination through induction of the EBV lytic cycle.^79^ For instance, supernatant from HONE1-EBV cells induced with suberanilohydroxamic acid could transduce 71% of Daudi cells in an EBV transduction assay but raised concerns with promoting viral dissemination.^80^ Indeed, a pilot study evaluating the efficacy and safety of romidepsin in treating extranodal natural killer/T-cell lymphoma reported a significant increase in viraemia among their patients.^81^ However, the novel compound C7^82–84^ and the antibiotic clofoctol^85^ were found to induce the expression of immediate-early and early-lytic proteins but not late-lytic proteins. Furthermore, these compounds did not lead to the production of EBV virions after lytic induction. Reactivating the EBV lytic cycle without producing virions makes them suitable candidates for lytic-induction therapy with minimal risk of viral dissemination.

### Subtype VI – common EBV mutations

EBV-positive tumours show several common mutations such as PI3K catalytic subunit alpha (PIK3CA; 80%), ARID1A (55%) and BCOR (23%) mutations,^86^ which have the potential to be treated by targeted therapy.

PI3K is an important tyrosine kinase inhibitor strongly associated with EBVaGC, which can bind to several ligands, including HER2 and EGFR.^87^ Mutations in exons 9 and 20 of the PIK3CA, as well as mutations in phosphatase and tensin homolog (PTEN), AKT1, AKT2 and AKT3, disrupt the PI3K–Akt–mTOR pathway and have been proposed as biomarkers for testing novel compounds and dosing schedules.^88^ Circular-RNA-AKT3 (circAKT3) has been linked to PIK3/Akt activation in GC and resistance to cisplatin.^89–91^ Cisplatin damages cancer cell DNA; therefore, by modulating the activation of the PI3K/AKT pathway, EBV enhances DNA repair mechanisms that may counteract the therapeutic effects of cisplatin. If this observation is confirmed in vivo, circAKT3 could serve as a promising prognostic marker for evaluating cisplatin resistance in patients with advanced GC. Several PI3K/Akt/mTOR inhibitors, such as idelalisib and copanlisib, have received approval from the FDA and are undergoing clinical testing.^92^ Everolimus, an mTOR inhibitor, demonstrated potential benefits in phase II trials for advanced GC^93,94^ but did not significantly improve OS in subsequent phase III trials.^95^ In a recent study by Chen et al.,^96^ the PI3K/mTOR dual inhibitor BEZ235 exhibited greater therapeutic efficacy than everolimus or the MEK inhibitor AZD6244 in paclitaxel-resistant GC cells.

ARID1A is also among the most frequently mutated genes in EBVaGC, suggesting that targeted therapies aimed at ARID1A mutations could offer substantial therapeutic benefits to patients.^97^ However, due to the frequent inactivation of the ARID1A tumour suppressor gene, ARID1A alone is not an effective therapeutic target. Additionally, potential candidate targets or pathways associated with ARID1A deficiency have yet to be identified, indicating that specific inhibitors targeting genes downstream of ARID1A deficiency may not provide therapeutic benefit.^98^ Recent advancements propose synthetic lethal approaches for treating ARID1A-deficient tumours using specific inhibitors against enhancers of zest-homolog-2 (EZH2).^99^ These strategies have demonstrated the selective sensitivity of EZH2 inhibitors against ARID1A-deficient GC cells, suggesting the potential efficacy of targeted therapy employing a synthetic lethal approach for ARID1A-deficient EBVaGC. Considering that treatment strategies based on synthetic lethality are effective in various cancers, Akt or PARP inhibitors are anticipated to exhibit efficacy in EBVaGC harbouring ARID1A mutations.^100,101^ These treatments targeting ARID1A-mutated or deficient tumours may be promising for EBVaGC management.

## Conclusion

This review is one of the first to identify therapeutic subtypes within the EBVaGC subtype. Through careful analyses of a wide range of studies from in vitro to clinical trials, we have identified six non-mutually exclusive EBVaGC subtypes and potential targeted treatments for each, summarised in Table 2. Each subtype has the potential to be treated with a specific strategy tailored for its characteristics, with the hope of improving OS and prognosis. These strategies range from ICI and antibiotics to ‘kick and kill’ and targeted inhibitors. While recognition of EBVaGC subtypes offers promising avenues for tailored therapy, the feasibility of routine subtype identification in clinical settings presents several challenges. Confirming EBV positivity typically requires in situ hybridisation for EBV-encoded RNA, whereas further stratification by immune checkpoint expression, MSI status or TMB requires immunohistochemistry and next-generation sequencing. Such multimodal testing may not be readily available at many institutions, and the high costs and long turnaround times required for this testing may delay treatment decisions. In addition, these subtypes are not mutually exclusive, which further complicates clinical decision-making. This calls for further studies and clinical trials examining potential therapeutic strategies for EBVaGC subtypes, which hopefully counteract the inconsistent results seen in immunotherapy treatment for EBVaGC and examine the cost-effectiveness and feasibility of these suggested clinical workflows.

**Table 2.** Summary of EBVaGC subtypes and therapeutic opportunities.

EBVaGC subtype	Characteristics	Therapeutic opportunities	Current research stage
Immune checkpoint expressing	Higher expression of PD-L1, CTLA-4 and TIM-3	ICIs	Phase III clinical trials
High microsatellite instability	Deficient mismatch repair complex	Immunotherapy, mainly ICIs	Phase III clinical trials
High tumour mutational burden	High number of somatic mutations and expression of neoantigens	Immunotherapy, mainly ICIs	In vitro studies
*Helicobacter pylori* infection	Inflammatory stress and early-onset GC	Antiviral agents and antimicrobials	In vitro studies
Wild-type p53	Co-activator of the BZLF1 gene expression	Lytic-induction therapy	In vitro studies
Commonly mutated genes	PI3K and ARID1A mutations	PI3K/mTOR dual inhibitor and Akt/PARP inhibitors	Phase II/III clinical trials in related cancers

EBVaGC, Epstein–Barr virus-associated gastric cancer; ICI, immune checkpoint inhibitor.

## Declarations

**Ethics approval and consent to participate:** Not applicable.

**Consent for publication:** Not applicable.

**Availability of data and materials:** Not applicable.
